# Environmental impact assessment in health technology assessment: principles, approaches, and challenges

**DOI:** 10.1017/S0266462323000041

**Published:** 2023-02-23

**Authors:** Michael Toolan, Sarah Walpole, Koonal Shah, Juliet Kenny, Páll Jónsson, Nick Crabb, Felix Greaves

**Affiliations:** 1 Department of Critical Care, Guy’s and St Thomas’ NHS Foundation Trust, London, UK; 2 Department of Infectious Diseases, Northumbria Healthcare NHS Foundation Trust, Northumberland, UK; 3 Faculty of Medical Sciences, Newcastle University, Newcastle upon Tyne, UK; 4 Science, Evidence and Analytics Directorate, National Institute for Health and Care Excellence, London, UK; 5 Department of Primary Care and Public Health, Imperial College London, London, UK

**Keywords:** health technology assessment, environmental sustainability, health policy

## Abstract

To reduce harm to the environment resulting from the production, use, and disposal of health technologies, there are different options for how health technology assessment (HTA) agencies can consider environmental information. We identified four approaches that HTA agencies can use to take environmental information into account in healthcare decision making and the challenges associated with each approach. Republishing data that is in the public domain or has been submitted to an HTA agency we term the “information conduit” approach. Analyzing and presenting environmental data separately from established health economic analyses is described as “parallel evaluation.” Integrating environmental impact into HTAs by identifying or creating new methods that allow clinical, financial, and environmental information to be combined in a single quantitative analysis is “integrated evaluation.” Finally, evidence synthesis and analysis of health technologies that are not expected to improve health-related outcomes but claim to have relative environmental benefits are termed “environment-focused evaluation.”

## Why account for environmental data when carrying out health technology assessment?

Manufacture, distribution, use, excretion, and disposal of health technologies all have environmental impacts. Domains of environmental impact include greenhouse gas emissions (GHGs); air, water, and soil pollution; depletion of resources; and solid waste production. Anthropogenic changes in all domains are threatening planetary and human health (1;2).

In 2021, 50 countries committed to developing sustainable, resilient health systems (3). Eighteen committed to achieving a net zero carbon health service (3). England’s target is net zero by 2045 for all services and products which the National Health Service (NHS) influences, including from procured health technologies (4). While GHGs are the current focus of international target setting, other areas of environmental impact are also important. For example, a medicine associated with few GHGs may cause significant ecotoxicity when discharged or excreted into the environment.

The aim of incorporating environmental considerations into health technology assessment (HTA) is to help optimize health system decision-making around technologies/interventions informed by information on environmental impact as well as clinical and cost outcomes. Depending on how HTA agencies choose to integrate environmental considerations into their work and their overall influence and remit, adapting HTA could also support wider efforts in health systems and society to reduce negative anthropogenic environmental impacts, with human and planetary health benefits now and in the future. Near-term benefits may include:

- helping to establish common standards for the quantification and reporting of environmental impact data of health technologies;
- promoting collection and disclosure of such data;
- incentivizing suppliers to develop more environmentally sustainable manufacturing techniques, products, and research practices.

Reducing the environmental impacts of health technologies will require collaboration between diverse stakeholders, including manufacturers, healthcare providers, patients, the public, governments, and regulators. The primary role of HTA agencies will be determining how best to use environmental data in value assessment and decision-making. In the following sections, we consider potential approaches HTA agencies could take.

## How can HTA agencies take environmental information into account?

We identify four approaches that HTA agencies can use to take environmental information into account in healthcare decision-making: information conduit, parallel evaluation, integrated evaluation, and environment-focused evaluation. Information conduit is distinct from the other options; under this approach, environmental considerations would not affect the HTA agency’s own value judgments. By contrast, parallel evaluation, integrated evaluation, and environment-focused evaluation are potential approaches for combining environmental impact data with other types of impact data (clinical or cost) in HTA decisions about value.

### Information Conduit

This approach involves an HTA agency republishing environmental data that is in the public domain or has been submitted to the HTA agency (e.g., by a manufacturer) without further assessment. HTA agencies present the information in a standardized format or simply reproduce it in the format that it was provided.

Potential benefits of this approach are that it need not be resource intensive for an HTA agency and it could promote the publishing and distribution of data on environmental impacts. This may facilitate more environmentally informed decision-making at other levels of the health system (e.g., by companies, clinicians, payers, or patients).

### Integrated Evaluation and Parallel Evaluation

Integrated evaluation involves fully integrating environmental impact into HTAs by identifying or creating new statistical methods or models that allow clinical, financial, and environmental information to be synthesized in a single quantitative analysis. Health economic approaches that could be adapted to this end include: cost-utility analysis factoring in environmental costs or outcomes; and cost–benefit analysis where environmental outcomes would be monetized (5).

Parallel evaluation is a more flexible approach to factoring environmental impact data into value judgments alongside clinical and cost outcomes. It entails an HTA agency analyzing and presenting environmental data alongside established health economic analyses. Results of the separate cost-effectiveness and environmental analyses could be presented to a decision-making panel to synthesize via a process of deliberation and consensus or via a multicriteria decision analysis type approach (6). Alternatively, results of the separate cost-effectiveness and environmental analyses could be subjected to completely different value frameworks (e.g., separate thresholds or criteria based on GHGs could be applied). Cost-effectiveness and environmental analyses could be assessed by different decision-making groups with the HTA agency.

Both integrated and parallel evaluation would require that HTA organizations place a value on environmental impacts relative to health impacts. Estimates of value and hence decisions about funding would be directly affected by the calculated environmental consequences, therefore, depending on the relative weightings applied to environmental outcomes as compared to health, both approaches are likely to be impactful in promoting environmental sustainability in healthcare procurement. The key theoretical benefit of separating analyses of health benefits and costs from environmental analyses (using parallel evaluation) is that the same assumptions or evidence standards would not be required in both types of analysis, giving more flexibility in the type of evidence which can be incorporated.

### Environment-Focused Evaluation

To date, HTA methods have evolved with the primary purpose of assessing the value of technologies that are expected to offer additional health or cost benefits compared to the standard of care. Environment-focused evaluation applies to technologies which are not usually assessed by HTA agencies: those not expected to improve health-related outcomes but claiming to have relative environmental benefits. Nitrous oxide capture and destruction technologies are an example: while not changing patient experience or outcomes these may reduce GHGs associated with nitrous oxide use (7).

Assuming that healthcare payers are willing to pay for environmental benefits without direct health benefits, evidence synthesis and analysis of the value of these technologies could inform purchasing decisions. Delivering this kind of assessment would require the creation of new decision-making frameworks. Such an approach could promote the development and implementation of healthcare technologies with primarily environmental benefits.

## What challenges do HTA agencies face?

### Developing Appropriate Analytical Techniques

Analytical techniques should be appropriate and proportionate in terms of complexity and cost to implement.

Technical challenges may be significant. Two important technical challenges are agreeing methods that can be applied across a variety of health technologies and standardizing quantification across different environmental domains (which vary in terms of maturity of quantification techniques and relative political and public prioritization).

Standardized and broadly accepted methods exist to quantify and value clinical and cost-effectiveness, but for environmental impacts such methods are in their infancy or altogether lacking. For biodiversity loss, for example, there is no agreed approach to quantification. Even where quantification methods exist, for example, for GHGs, granular and specific data are lacking since such impacts are not yet reported by manufacturers, in clinical trial outputs or in submissions to regulators and HTA agencies.

There are important questions of analytical scope (boundaries) that HTA agencies may play a role in resolving, depending on the approach they take. These include:

- domains of environmental impacts considered (including species and ecosystems considered) and weighting of impacts relative to each other in analyses;
- analytical perspective (e.g., health system or societal, national or global), which costs and outcomes across which areas (e.g., health sector, wider society, other nations, environment) are included and how (and how much) they are valued (5);
- time horizon over which impacts are measured, forecast, or discounted (e.g., are impacts on future generations included, and if so, how are they weighted against impacts affecting current populations?);
- parts of a technology’s lifecycle to which impacts relate (production, distribution, use, or disposal) and whether both “direct” and “indirect” impacts should be included (e.g., indirect impacts that arise at the use stage include the downstream environmental consequences of the technology’s impact on the disease course and subsequent healthcare resource utilization (6)).

An important question is how to approach trade-offs between financial costs, health outcomes, and environmental outcomes when technologies improve some of these but worsen others. Achieving societal consensus on this will be challenging.

If environmental impact data is to feed directly into the HTA value decision (as is the case with integrated evaluation, parallel evaluation, and environment-focused evaluation), HTA agencies will want to understand societal preferences, specifically how much people are willing to pay, monetarily or in forgone health, for environmental gains. As this is not yet known, resource would be required to investigate people’s preferences. It is worth nothing that lack of empirical evidence on societal willingness-to-pay (8) has not prevented NICE in England from establishing a cost-effectiveness threshold on which to base its current analyses methods and value framework. NICE’s threshold has come under considerable scrutiny (8;9) but has, to date, remained in use. An additional question for parallel and integrated evaluation is whether environmental factors should be included as costs or outcomes (10).

Adopting the information conduit approach would allow HTA agencies to avoid the work of exploring societal preferences themselves. This approach, however, is likely to have minimal impact in the absence of a framework (whether produced by HTA agencies themselves, government or another competent agency) prescribing how data should be used by the health sector in its decision-making.

### Achieving Consensus on How to Balance Environmental Impacts against Other Costs and Benefits

Use of any health technology may incur costs and have outcomes that fall outside the health sector. Some HTA agencies take a broader societal perspective while others have a narrower health sector focus. Which perspective is taken affects how the objectives of the health sector should be balanced against other public sector expenditure.

In the health economics literature upon which much current HTA practice is based, the environment is typically characterized as a sector of the economy. The discipline of ecological economics presents an alternative paradigm whereby the human economy is viewed as a subsystem of a larger ecosystem (10). This suggests that environmental impacts should be treated differently from other wider costs and outcomes because all human outcomes (health or otherwise) are subsidiary to and dependent on planetary health.

Recent papers discuss the application of ecological economic theory to sustainable healthcare decision making (10–12). Hensher argues that health economists can no longer afford to solely occupy themselves with refining methods related to achieving allocative efficiency, they must also consider the scale of the economy and whether it can be sustained (10). Doing so may render some of the previous literature on analysis perspectives redundant (e.g., (13)). To deal with methodological challenges related to perspective, HTA agencies will have to overcome the embedded challenge of updating health economics scholarship to align with current environmental science and macroeconomic theory.

### Directing Analytical Skills and Resources to the Task

HTA agencies and the life sciences industry must be willing to develop their methodological acumen, complete new types of analyses, and act on results. This entails training existing staff and collaborators such as academic groups and consultancies, recruiting new staff with relevant environmental and economics expertise, and funding staff time to complete assessments.

If value frameworks are updated or new ones are created, HTA decision-making panels will require new skills to interpret and critique environmental data. If panels are required to arrive at a single recommendation via a deliberative process drawing on clinical, cost, and environmental inputs, they will need guidance on how to weight different impacts in discussions and how to explain their reasoning transparently.

To mitigate the resource burden, detailed environmental analyses could be carried out contingent on gating criteria, so they are focused on higher impact topics, not applied uniformly to all health technologies. This would likely come with a short-term resource burden of working out the appropriate gating criteria.

### Addressing the Consequences of Implementation

Incorporating environmental considerations in HTA may involve operational and political risks, unintended consequences and opportunity costs affecting patients, the life sciences industry, and the health system. Certain manufacturers’ profits may be disproportionately affected by revised HTA value frameworks. At worst, this could result in patients losing access to certain products. Alternatively, if HTA agencies adopt a light touch information conduit approach to avoid confrontation with the life sciences industry and save their own resources, the risk of bias inherent in this approach may result in procurement becoming even less environmentally friendly and therefore harmful to humans and the economy than it is currently. HTA agencies may anticipate and monitor for such risks, consequences, and opportunity costs, which may inform which approach they employ to take environmental information into account, in which circumstances, and how they communicate about this with stakeholders.

## Implications for HTA agencies and health systems

Existing HTA processes could be adapted to incorporate environmental information, encouraging or mandating manufacturers to provide such information. Transparency about environmental costs or outcomes could inform decision-making by HTA agencies themselves and other actors. It may motivate manufacturers to reduce environmental impacts across the lifecycle (14).

The approaches to incorporating environmental data in HTA outlined above may have different roles and be applied at different times. An HTA agency could, for example, apply information conduit, parallel evaluation, and integrated assessment sequentially based on gating criteria (depending on expected environmental impacts or data availability).

Two HTA agencies have made strategic commitments to address environmental sustainability (15;16). Canada’s agency has adopted a value framework for evaluating non-drug health technologies which allows for deliberative consideration of environmental effects, consistent with the parallel approach described here (17). HTA agencies must invest in developing or hiring relevant expertise to carry out environmental assessments and incorporate environmental data in health economic methodologies. The greatest challenge may be achieving consensus on how to balance environmental impacts against other valued costs and benefits.
